# New Paradigm in Diabetic Foot Ulcer Grafting Techniques Using 3D-Bioprinted Autologous Minimally Manipulated Homologous Adipose Tissue (3D-AMHAT) with Fibrin Gel Acting as a Biodegradable Scaffold

**DOI:** 10.3390/gels9010066

**Published:** 2023-01-13

**Authors:** Mohd Yazid Bajuri, Jeehee Kim, Yeongseo Yu, Muhammad Shazwan Shahul Hameed

**Affiliations:** 1 Department of Orthopaedics and Traumatology, Faculty of Medicine, National University of Malaysia, Kuala Lumpur 56000, Malaysia; 2 ROKIT AMERICA, 3580 Wilshire Blvd., 900-27, Los Angeles, CA 90010, USA

**Keywords:** 3D bioprinter, autologous adipose tissue graft, diabetic foot ulcer, fibrin glue, biodegradable scaffold, tissue regeneration

## Abstract

Adipose tissue is an abundant source of extracellular substances that support the tissue repair process. This pilot study was carried out to determine the efficacy of 3D-bioprinted autologous adipose tissue grafts on diabetic foot ulcers (DFUs), with fibrin gel used to stabilise the graft. This was a single-arm pilot study in a tertiary hospital that provides diabetic wound care services. A total of 10 patients with a DFU were enrolled, and the primary endpoint was complete healing within 12 weeks. The secondary endpoints were wound size reduction, time to healing, and adverse events. Seven out of ten patients showed complete healing of their DFU within 12 weeks (at 2, 4, 5, 10, and 12 weeks, respectively). The wound size reduction rate was significantly and progressively reduced over time. According to our data, autologous adipose tissue grafting using a 3D bioprinter, with the addition of fibrin gel that acts as a scaffold, promotes wound healing with high-quality skin reconstruction. Throughout this study period, no adverse events were observed.

## 1. Introduction

Diabetes mellitus is a major non-communicable illness that affects millions of people worldwide [1]. Long-term uncontrolled high blood sugar levels frequently result in neuropathy and peripheral vascular disease, which can lead to a plethora of problems, including diabetic foot ulcers (DFUs) [2]. Three out of every twenty diabetic patients are affected by DFUs, facing greater risk of disability via amputation or even death [3]. This undoubtedly exposes the DFU patients and their families to significant financial burden that may arise from the disease.

Blood sugar monitoring, wound debridement, moist dressings, antibiotic therapy for wound infection, and weight-bearing ulcer offloading are all part of conventional DFU care [4]. However, these methods fail to resolve the ulcers completely, and patients are often left with recurring DFUs, which greatly affect their quality of life [2]. The emergence of skin substitute technology has changed the paradigm of DFU treatment [5]. Accordingly, a variety of biological skin substitutes, derived from either natural or synthetic biomaterials, have been developed over the last 20 years to improve the prognosis of DFUs [5].

Mojallal et al. paved the way for the use of autologous fat grafting in wound healing through their observation of enhancement in collagen fibre neosynthesis, vascularisation, and the thickness of the dermis and subcutaneous tissue with this easily accessible tissue source [6]. Since then, evidence supporting the efficacy of autologous fat grafting in wound healing has continued to grow over time [7,8].

In recent years, 3D bioprinting has emerged as a crucial tool that brings a great deal of flexibility and versatility, particularly in the fabrication of biological skin substitutes, due to the ability to customise them according to the need of the wound [9]. Using computer scanning imaging systems, 3D bioprinting technology enables the fabrication of skin substitutes that match the size and depth of the wound. A suitable bioprinting material should be biocompatible and easily manipulated to be dispensed in complex 3D structures while still maintaining its viability and function [10].

The skin substitutes constructed using biomaterials compatible with the human body possess interconnected pores and large surface areas that can support cell attachment, proliferation, and growth, greatly improving the quality of the engineered skin graft. Furthermore, various biological agents such as growth factors and nucleic acids can be deposited, which further enhance the wound healing process [9,11]. Numerous processing techniques, including the most recent 3D printing technology, provide additional tools to tailor the hydrogel properties to specific applications [12]. Fibrin gel is a biological tissue adhesive that mimics the last steps of the coagulation cascade that occurs when thrombin activates a solution of human fibrinogen.

Fibrin gel was used to create a scaffold, as it exhibits minimal inflammation and foreign body reaction and is readily absorbed during the normal wound healing process [13]. The scaffold must be sturdy enough to withstand mechanical stress from the surrounding tissue, since mechanical strength is a crucial component in its development. On the other hand, changes in the scaffold’s size may be impacted by its poor mechanical strength [14].

Therefore, this study aims to ascertain the healing effects of 3D-bioprinted autologous minimally manipulated homologous adipose tissue (3D-AMHAT) on DFUs using both autologous adipose tissue transplants and 3D bioprinter technology with fibrin glue as a biodegradable scaffold.

## 2. Results

### 2.1. Participants

Screening for inclusion was conducted for 10 patients during the study period between August and December 2021. Following assessment of eligibility criteria, all 10 patients were included in the study. The baseline demographic and clinical characteristics of the participants are summarised in Table 1.

### 2.2. Complete Epithelialisation of the Wound

**Figure 1:**
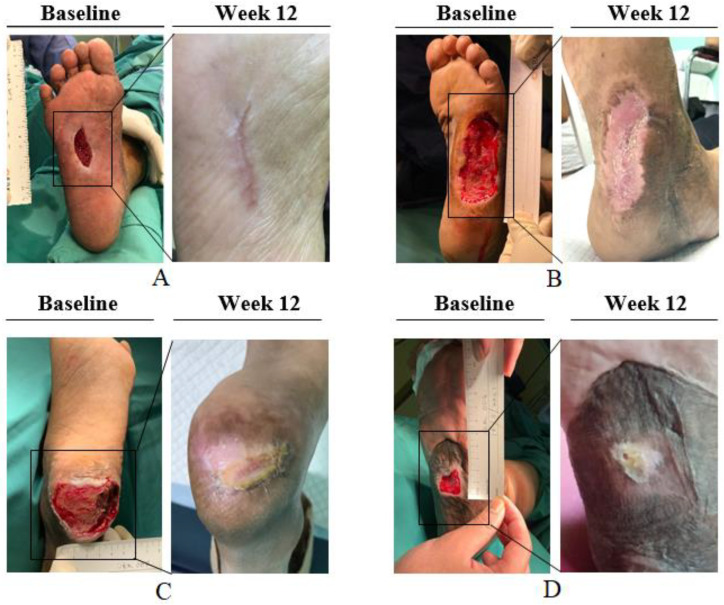
Complete wound healing outcomes among the patients with diabetic foot ulcers: (**A**–**D**) Complete healing of 4 patients within 12 weeks. Black squares at baseline indicate the ulcer before the treatment. Complete wound healing images at week 12 are magnified.

Complete healing of the ulcer with 100% epithelialisation was observed in 7 out of 10 patients within 12 weeks. The remaining patients demonstrated ulcer healing of over 80% at week 12 (Figure 1).

### 2.3. Wound Area Reduction

**Figure 2:**
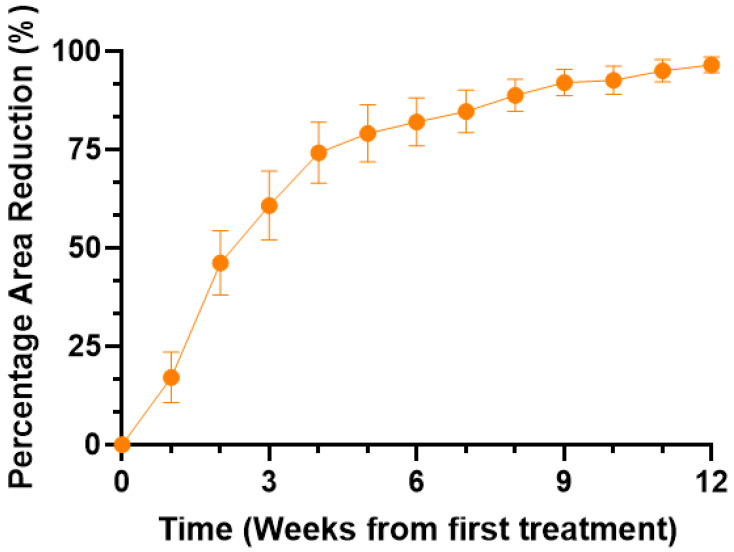
Average percentage wound area during the course of the study (*n* = 10). Repeated measures one-way ANOVA revealed a significant trend of increasing reduction (*p* < 0.05).

**Figure 3:**
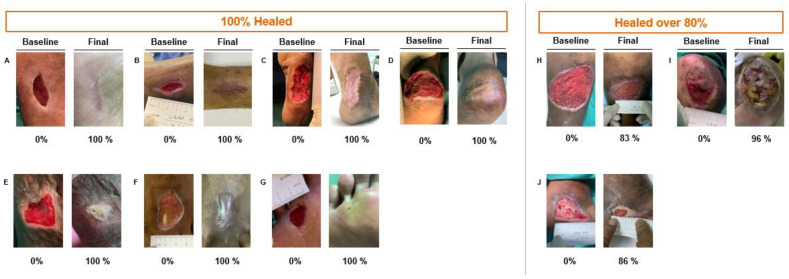
Corresponding wound pictures taken from the study (*n* = 10) (**A**–**J**).

The data plot of weekly percentage reduction in the wound area depicted in Figure 2, along with the corresponding pictures in Figure 3, reveal a significant (*p* < 0.05) progressive reduction in the wound size and/or volume.

### 2.4. Time to Healing

**Figure 4:**
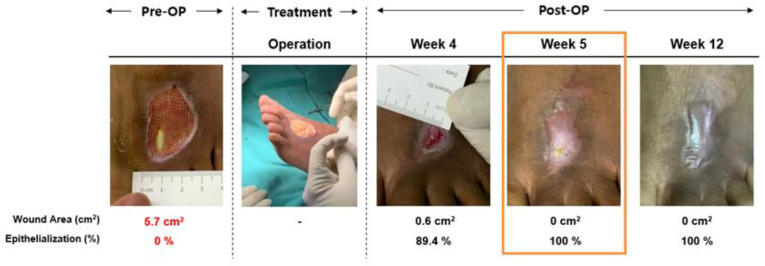
Wound healing process in a patient with an exposed tendon using 3D-AMHAT. The patient’s wound healed completely after 5 weeks of using 3D-AMHAT.

The fastest complete wound healing was observed at week 2 in one patient. Overall, 7 out of 10 patients showed complete healing of their DFU within 12 weeks (in 2, 4, 5, 10, and 12 weeks, respectively). The wound size reduction rate was significantly and progressively reduced over time. There was no evidence of keloid formation, scarring, adjacent tissue contracture, or infection. The healed ulcers appeared similar to the surrounding native tissue. One example that we saw in our case was that there was a good outcome in one of the patients who had an exposed tendon, where there was no contracture over the wound which causing the skin to be tethered. Furthermore, the tendon was able to glide smoothly over the area. It was observed that when we used the 3D-AMHAT technique, there was no skin adherence seen and the wound healed with better quality, as shown in Figure 4.

### 2.5. Discussion

This study reports for the first time the application of 3D-bioprinted autologous minimally manipulated homologous adipose tissue (3D-AMHAT) for diabetic foot ulcers (DFUs). The observed outcome of the current study shows that applying the 3D-AMHAT to the DFU area increased the rate of wound healing, which is otherwise slow if left untreated [4]. Untreated DFUs usually show significantly less and slower wound reduction compared to treated DFUs when measured every 10 days [15]. These observations support the purpose of this study, which was to assess the efficacy of 3D-AMHAT with fibrin glue acting as a biodegradable scaffold in enhancing the rate of healing of chronic wounds to the lower extremities in patients with diabetes mellitus.

All ulcers demonstrated complete epithelialisation during the 12-week period of the study except, for three, which still showed an over 80% decrease in the ulcer size. This suggests that a longer follow-up period may reveal a higher rate of complete healing compared to the present study. According to the Malaysian clinical practice guidelines for management of diabetic feet, an average 3–6 months of follow-up is required for the monitoring of healing of moderate-risk DFUs [16]. Indeed, a further follow-up with the slowest-healing patient revealed complete healing at week 17. Nevertheless, considering the chronic nature of DFUs, healing of over 80% after 12 weeks of treatment with 3D-AMHAT suggests the efficacy of this technology in treating this condition. Furthermore, DFU patients with smaller and milder ulcers are known to have a better prognosis compared to patients with larger and more severe ulcers [17]. This applies to treatments for DFUs other than 3D-AMHAT, such as hyperbaric oxygen therapy (HBOT) [18,19]. Overall, our outcomes are positive for the continued validation of the 3D-AMHAT technology for subsequent phases of clinical trials.

The constant and significantly progressive reduction in ulcer size observed in the present study is consistent with the observations made by Chopinaud et al. when treating hypertensive leg ulcers with autologous adipose tissue grafts [7]. Steady progression of the wound healing contributes greatly to the quality of the healed skin, improving the quality of life of DFU patients [20]. This is because excessive or rapid rates of healing may cause the formation of fibrous scar tissue [21]. Utilising a diabetic rat model to evaluate the quality of the healed skin following autologous nanofat transplantation, Chen et al. successfully demonstrated a greater regenerated skin quality with adipose tissue grafts [22]. Accordingly, treatment of DFUs with 3D-AMHAT may be advantageous in the context of the quality of the healed skin without contracture. In the zone of healed ulcers, there were also no signs of erosion. Enhanced by the 3D printing technology, AMHAT can be engineered in a way that matches the size and depth of the ulcer wound using computer scanning imaging technology. Different biological agents, including living cells, growth factors, and nucleic acids, can be deposited to mimic the morphology and physiology of normal human skin. Moreover, the technology also enables the construction of 3D-bioprinted skin grafts with interconnected pores and large surface areas that support cell attachment, growth, intercellular communication, and exchange of gases and nutrients. All of these properties improve the quality of AMHAT constructs and promote the healing of ulcerous wounds [9].

The time to healing outcome in the present study revealed a diverse pattern of the time taken for different patients to achieve complete healing. Time to healing is a parameter that depends on mainly on the initial size or depth of the wound. Additionally, personal factors such as age also play a role in the wound healing rate which leads to different outcomes for time taken to heal [17]. Diverse patterns of time to healing with adipose tissue grafts were also reported in previous studies [7,8]. When the patients’ demographics and initial wound size were examined, the time to healing was indeed affected by these factors, whereby younger patients with smaller initial wounds healed sooner than older patients with larger initial wounds. This finding is consistent with that reported in another study, which showed that patients with longstanding chronic DFUs experienced poorer healing compared to those with recently developed DFUs when receiving the same treatment protocol [15].

The absence of any adverse effects observed among the participants of the present study is consistent with the established safety of using adipose tissue grafts for the treatment of DFUs, as reported by numerous previous studies [6,7,8]. After the wound healing process, one of the common complications that requires additional surgery is scar tissue contracture. We were able to conclude that there was an absence of scar and adjacent tissue contracture. In many skin substitute therapies, the adverse effects reported are typically associated with the extensive manipulation of the biomaterial ex vivo [22]. Accordingly, the 3D-AMHAT technology applied in the present study offers an extra layer of assurance by reducing the extensive manipulation of the tissue following liposuction. Essentially, microbial infections due to tissue contamination can also be prevented following filtration and dispensing of the harvested adipose tissue using the function equipped by the 3D bioprinter.

Due to the exploratory nature of this study, an obvious limitation exists in the context of comparing the efficacy of 3D-AMHAT in enhancing wound healing against that of other common treatments for DFUs, such as amnion grafts and skin substitutes. Other limitations include our lack of a control group, lack of long-term follow-up for more than 12 weeks for the occurrence of side effects and incidence of relapses, and small sample size. However, this is mitigated by the fact that the principal purpose of the study was achieved, in that the procedure was shown to be safe since there were no procedure-related side effects, and no significant adverse events related to the cells were reported during the course of the follow-up. The findings of the present study could be supplemented by comparison of 3D-AMHAT’s efficacy in enhancing diabetic wound healing against other therapies for DFUs in the future.

## 3. Conclusions

The 3D-bioprinted autologous minimally manipulated homologous adipose tissue (3D-AMHAT) with the addition of fibrin gel as a scaffold is a potentially effective treatment method for improving skin regeneration in patients with DFUs. Further validation with subsequent phases of clinical trials is feasible and paramount for this therapy.

## 4. Materials and Methods

### 4.1. Study Design

This study was a single-arm pilot study to assess the efficacy of 3D-bioprinted autologous minimally manipulated homologous adipose tissue (3D-AMHAT) with the addition of fibrin gel to enhance the wound healing in a population of diabetic foot ulcer patients. The study was approved by the Universiti Kebangsaan Malaysia Research Ethics Committee (UKMREC) with approval number UKM PPI/111/8/JEP-2021-285. The study was conducted in accordance with the Declaration of Helsinki and the Malaysian Guidelines for Good Clinical Practice. The study was supported by a grant from Universiti Kebangsaan Malaysia. ROKIT Healthcare Inc. provided the Dr. INVIVO clean chamber 3D bioprinter and the Dr. INVIVO AI Regen KIT disposable bioprinting supplies for this study free of cost.

### 4.2. Participants

The study included patients attending the Diabetic Foot Wound Care and Reconstruction Clinic at the Orthopaedics and Traumatology Department of Hospital Canselor Tuanku Muhriz in Kuala Lumpur, Malaysia. All patients were carefully counselled by the investigating team, and informed consent was obtained. The consent form had been previously approved by the Ethics Committee.

### 4.3. Inclusion Criteria

The study included patients from both sexes between 30 and 64 years of age with established wounds to their lower extremities as a result of type I or type II diabetes mellitus. Additionally, the included patients also presented with transcutaneous oxygen pressure above 40 mmHg or a detectable foot pulse by the Doppler test. Finally, only patients who were able and willing to consent and comply with the study protocols were included in the study.

### 4.4. Exclusion Criteria

Patients were excluded in the event of the patients having an ulcer located over a Charcot deformity; presenting with clinical signs of inflammation or infection in the ulcer area; being diagnosed with a malignant tumour; having a history of participating in another clinical study within 4 weeks; taking either systemic corticosteroids, immunosuppressants, or cytotoxic agents; and any other reason for which the patient was deemed to be unsuitable for the study by the principal investigator.

### 4.5. Intervention

**Figure 5:**
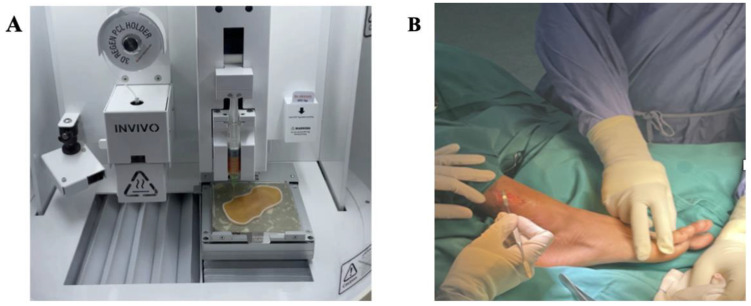
Photograph of the procedure: (**A**) Dispensing of fat tissue according to the wound size. (**B**) Application of the dispensed 3D-AMHAT onto the wound.

All patients were treated with a single application of 3D-AMHAT on the ulcer. Following recruitment into the study, each patient attended a treatment visit, where a liposuction procedure was conducted under local anaesthesia to harvest 15 to 25 mL of the patient’s fat tissue. The harvested fat was filtered and dispensed by the Dr. INVIVO clean chamber 3D bioprinter (ROKIT Healthcare, South Korea) to maintain the minimal manipulation. The dimensions of the dispensed AMHAT were set according to a predetermined size of the wound and combined with fibrin gel to create a scaffold using computer scanning imaging technology before the procedure (Figure 5A). The dispensed AMHAT was then transferred onto the wound by the investigator (Figure 5B). The 3D-AMHAT was then covered with a primary wound dressing, such as Mepitel^®^, and a secondary wound dressing, such as foam dressing.

### 4.6. Follow-Up

All patients were assessed weekly for up to 12 weeks following the application of 3D-AMHAT. At each follow-up visit, the surface area of the ulcer was measured and photographs were documented. Routine vital signs were also documented for pharmacovigilance reporting.

### 4.7. Outcome Measures and Data Analysis

The primary endpoint of the study was the complete epithelialisation of the wound within the 12 weeks of follow-up after treatment. Secondary endpoints were analysed from the wound measurements at each follow-up visit, including the wound size and/or volume reduction, along with the time to healing. Repeated measures one-way ANOVA was conducted for the wound size and/or volume reduction, while the time to healing function was analysed with Kaplan–Meier survival analysis using SPSS ver. 22 (IBM, Armonk, NY, USA). A *p*-value of <0.05 was considered statistically significant for the one-way ANOVA.

## Figures and Tables

**Table 1:** The baseline demographic and clinical characteristics of the participants.

Total Number of Patients (*n*)	10
Age in years (mean ± SD)	48.7 ± 14.9
***Gender***	***Number of patients (%)***
Male	7 (70%)
Female	3 (30%)
***Presence of comorbidities***	***Number of patients (%)***
1 comorbidity	4 (40%)
>1 comorbidities	6 (60%)
***Types of comorbidities***	***Number of patients (n)***
Type II diabetes mellitus	9
Hypertension	5
Dyslipidaemia	2
Spondylodiscitis	1
End-stage renal failure (ESRF)	1
Ankle–Brachial Pressure Index (ABPI) (mean ± SD)	1.11 ± 0.24
Baseline ulcer size at week 0 in area (cm^2^) (mean ± SD)	27.00 ± 43.99

## Data Availability

Not applicable.
